# Does a kidney stone culture make sense? The findings of microbiological cultures of kidney stones and correlation with stone composition, preoperative urine testing and postoperative SIRS

**DOI:** 10.1007/s00345-025-05772-5

**Published:** 2025-06-25

**Authors:** Hugo W. Schuil, J. van de Kamp, T. R. de Weijer, E. P. M. van Elzakker, J. Baard, A. C. Bouma-Houwert, M. M. E. L. Henderickx, C. A. Goossens-Laan, J. A. van der Spruit, R. J. A. van Moorselaar, H. P. Beerlage, B. M. A. Schout, G. M. Kamphuis

**Affiliations:** 1 Department of Urology, Amsterdam UMC, University of Amsterdam, De Boelelaan 1117, Amsterdam, 1081 HV The Netherlands; 2 Cancer Center Amsterdam, Amsterdam UMC, Amsterdam, The Netherlands; 3 Department of Urology, Alrijne Hospital, Leiderdorp, The Netherlands; 4 Department of Urology, Amsterdam UMC, Vrije Universiteit, Amsterdam, The Netherlands; 5 Department of Medical Microbiology and Infection Prevention, Amsterdam UMC, Amsterdam, The Netherlands

**Keywords:** Kidney stone cultures, Urolithiasis, Percutaneous nephrolithotomy, Infectious complications, Stone composition

## Abstract

**Purpose:**

The study investigates the results of kidney stone cultures and its correlation with preoperative urine tests, stone composition and postoperative systemic inflammatory response syndrome (SIRS) in patients undergoing percutaneous nephrolithotomy (PCNL).

**Methods:**

Data from 338 PCNL procedures performed between January 2018 and September 2023 at two centers in the Netherlands were included for analysis. Preoperative urine tests, kidney stone analysis and the outcome of kidney stone cultures were evaluated in addition to general patient characteristics and surgical information. Preoperative urine tests and stone cultures were concordant if they were both negative or both positive with the same microorganism. Multivariable logistic regression evaluated factors associated with positive stone cultures and their relationship to postoperative SIRS.

**Results:**

Stone cultures were positive in 43% of cases and were concordant with preoperative urine tests in 66% of cases. Stone culture results identified 33 different microorganisms, extending beyond urease-positive bacteria: most commonly found were *Enterococcus faecalis*, *Escherichia coli*, and *Proteus mirabilis*. Positive stone cultures were found across all types of stone composition. Positive stone cultures were significantly associated with postoperative SIRS (OR 3.12, 95% CI 1.01–9.65) and a positive preoperative urine test was not (OR 1.60, 95% CI 0.56–4.53).

**Conclusion:**

Stone cultures provide additional microbiological insights to preoperative urine testing. All types of stone composition can be associated with positive stone cultures, questioning the current thinking of infection vs. non-infection stones.

## Introduction

A central issue in the treatment of urolithiasis is the relationship between microorganisms and kidney stone formation. Urinary tract infections can contribute to the development of kidney stones, while conversely, obstructive kidney stones can lead to infection of the urinary tract [1]. Moreover, kidney stone surgery carries a serious risk of infectious complications. The European Association of Urology (EAU) guidelines therefore recommend preoperative urine testing and perioperative antibiotic prophylaxis for all kidney stone surgery [2]. 

Despite the use of antibiotics, systemic inflammatory response syndrome (SIRS) develops after 6.3% of ureterorenoscopy (URS) cases and 13% of percutaneous nephrolithotomy (PCNL) cases. Infectious complications can lead to prolonged hospitalization, extended recovery periods and mortality [3–5]. 

To better understand microorganisms of the urinary tract and urolithiasis, urine and kidney stones can be send for culture and stone analysis [2]. However, stone cultures are poorly studied, and while kidney stones are classified as infection stones or non-infection stones, the relationship between stone types and microorganisms remains unclear [6]. Furthermore, stone cultures are advised in the EAU guideline, but are rarely included in standard perioperative protocols [7]. 

This study presents findings on the prevalence and spectrum of microorganisms identified in stone cultures from kidney stone surgery. It examines the relationship between preoperative urine test results and stone culture outcomes, explores associations between stone composition and culture findings, and analyzes how urine and stone culture results correlate with the risk of postoperative SIRS.

## Methods

Consecutive percutaneous nephrolithotomy (PCNL) procedures in adults (≥ 18 years) at two medical centers in the Netherlands—Amsterdam UMC, Amsterdam, the Netherlands (inclusion between January 2018 and September 2023) and Alrijne Ziekenhuis, Leiderdorp, the Netherlands (inclusion between January 2019 and September 2023)—were identified through a search of the electronic health records.

Preoperatively, patients’ midstream urine was tested using a urine strip. If the strip indicated the presence of leukocytes or nitrites, the urine sample was sent for culture. Additionally, urine was directly sent for culture for patients with a nephrostomy tube, bladder catheter, JJ stent, a history of urinary tract infections, or those considered frail by the treating urologist. Urine cultures were processed according to the local standard operation procedures, based on the clinical microbiology procedures handbook. Urine cultures were deemed negative if no relevant growth of uropathogens or contamination was seen [8, 9]. 

All patients received perioperative prophylactic antibiotics according to local protocols, typically as a single intraoperative dose in case of a negative preoperative urine test. In case of positive urine culture, patient frailty, or the presence of indwelling catheters, antibiotics were initiated preoperatively and administered in a therapeutic dose and duration in accordance with the results and possible antibiogram of the preoperative urine culture, with a preference for ciprofloxacin and trimethoprim/sulfamethoxazole.

Preoperative physical status was assessed by an anesthesiologist using the American Society of Anesthesiologists (ASA) physical status classification system [10]. Patients underwent preoperative CT scans on which the expected complexity of the procedures was assessed using the Guy’s Stone Score [11]. Routine ureteral pre-stenting was not performed. Procedures were carried out by one or two experienced endourologists. PCNL was often combined with URS in endoscopic combined intrarenal surgery (ECIRS). Renal access was achieved under guidance from ultrasound, X-ray, and, when possible, endoscopic visualization. Postoperative management included the use of a double-J ureteral stent (JJ), a nephrostomy tube (NT), or a combination of both as exit strategies.

During PCNL, stone fragments were collected directly from the Amplatz sheath on a sterile gauze and placed in a container which was sent to the microbiology laboratory. The entire specimen was then inoculated in a brain heart infusion (Amsterdam UMC) or thioglycolate broth (Alrijne) and then assessed for five to seven days for signs of growth. If this growth was present, subculture was performed onto blood agar plates. These plates were incubated aerobically and anaerobically to identify the microorganism. Due to this method of culturing, a number of colony-forming units cannot be reliably presented and any growth was considered a positive culture. In case of polymicrobial cultures all microorganisms were presented.

Additionally, stone fragments were sent for clinical composition analysis. Stone analysis was performed by X-ray diffraction, Fourier Transform Infrared Spectroscopy (FT-IR) (Spectrum Two FT-IR machine (PerkinElmer, Waltham, USA) or Shimadzu IRAffinity-1 S (Taawon, Amman, Jordan)). All components of composition analysis were charted and classified according to the EAU guideline [2]. 

Fever was defined as a body temperature > 38.0 °C in at least two measurements. Postoperative SIRS was defined by any two of the following criteria: body temperature > 38.0 or < 36.0 °C; heart rate > 90 beats/minute; respiratory rate > 20 breaths/minute or Leukocyte count > 12,000 or < 4000/microliters [5]. 

Multivariable logistic regression analysis was performed to assess whether stone cultures were independently associated with postoperative SIRS, using both urine testing and stone culture results as independent variables. In a separate analysis, multivariable logistic regression was conducted to identify factors associated with positive stone cultures. Independent variables were selected based on existing literature on microbial presence and postoperative infections in the urinary tract, and included age, gender, ASA score, recurrent urinary tract infections, previous ipsilateral stone surgery, positive preoperative urine culture, GSS, preoperative nephrostomy, and preoperative JJ stent [12–14]. 

## Results

During the inclusion period, 429 PCNL were performed: 333 procedures were performed at Amsterdam UMC and 96 at Alrijne Ziekenhuis. Five patients objected to the use of their data, resulting in 424 procedures of which data was collected. Stone cultures were obtained in 80% of these procedures, these 338 were included for analysis in the present study.

Baseline characteristics are summarized in Table 1. The patients had a mean age of 56 years (SD 15), ASA scores were distributed ASA-I 13%, ASA-II 61%, ASA-III 25%, ASA-IV 0.9% and 18% presented with anatomical variations. A history of urinary tract infections was reported by the treating urologist in 38% of patients, while 54% had undergone previous urinary tract surgery for urolithiasis. The mean size of the largest stone was 24 mm, with a median of 2 (IQR 1–4) stones. The Guy’s Stone Score distribution was 14%, 38%, 25%, and 24% for grades 1, 2, 3, and 4, respectively. Urine was sent for culture at a median of 9 days (IQR 7–16 days) before surgery. ECIRS was performed in 71% of procedures. The mean procedure time was 111 min.

**Table 1:** Patient, stone and treatment characteristics, *N* = 338

	*N* (%)	Mean (SD)	Median (IQR)
Age		56 (15)	
Female	155 (46%)		
BMI		28 (6.1)	
ASA-score			
I	45 (13%)		
II	206 (61%)		
III	84 (25%)		
IV	3 (0.9%)		
Guy’s stone score (GSS)			
I	47 (14%)		
II	127 (38%)		
III	83 (25%)		
IV	81 (24%)		
Urinary tract abnormalities	60 (18%)		
Anatomical variation	20 (5.9%)		
Reconstructed lower tract	20 (5.9%)		
Reconstructed upper tract	3 (0.9%)		
Solitary kidney	11 (3.3%)		
Transplanted kidney	6 (1.8%)		
Diameter of largest stone (mm)		24 (13)	
Number of stones			2 (1–4)
Radio density of largest stone (HU)			
0–599	69 (20%)		
600–999	95 (28%)		
> 1000	169 (51%)		
Missing	5 (1.5%)		
Previous stone surgery	164 (54%)		
On the same side	143 (47%)		
Duration of surgery (min)		111 (41)	
PCNL/mini-PCNL			
Mini-PCNL (12–20 French)	193 (57%)		
Standard PCNL (21–30 French)	135 (40%)		
Missing	10 (3.0%)		
Combined with URS (ECIRS)	239 (71%)		
Exit strategy			
JJ stent	261 (77%)		
NT	108 (32%)		
JJ stent and NT	31 (9.2%)		
Endoscopic stone free	268 (79%)		
Radiological stone free (no fragments > 4 mm on NCCT)	150 (44%)		
Secondary treatment indicated	102 (30%)		
Post-operative complications in Clavien-Dindo (%)			
None	249 (74%)		
CD-I	22 (6.5%)		
CD-II	53 (16%)		
CD-IIIa	1 (0.3%)		
CD-IIIb	9 (2.7%)		
CD-IV	3 (0.9%)		
CD-V	1 (0.3%)		
Fever	54 (16%)		
SIRS	23 (6.8%)		
Blood transfusion	16 (4.7%)		
Coiling	3 (0.9%)		
Postoperative hospital stay (days)			1 (1–3)

*BMI* Body Mass Index, *ASA* American Society of Anesthesiologists, *GSS* Guy’s Stone Score, *HU* Hounsfield Units, *PCNL* Percutaneous Nephrolithotomy, *ECIRS* Endoscopic Combined Intra-Renal Surgery, *URS* ureterorenoscopy, *JJ* Double-J stent, *NT* Nephrostomy Tube, *NCCT* Non-Contrast Computed Tomography, SIRS: Systemic Inflammatory Response Syndrome

Perioperative antibiotic prophylaxis was administered in 330 procedures (98%), primarily as a single dose given immediately before surgery. In 146 procedures (43%), this prophylactic dose was the only antibiotic used. Cefuroxime (*n* = 228, 67%) and gentamicin (*n* = 81, 24%) were the most frequently administered agents, often in combination. In 147 procedures (43%), therapeutic-dose antibiotics were initiated before surgery, with a mean preoperative duration of 5.2 days (SD 8.3). The most commonly used agents were ciprofloxacin (*n* = 58, 17%) and trimethoprim/sulfamethoxazole (*n* = 40, 12%). Postoperatively, antibiotic treatment was continued in 154 procedures (46%) for a mean duration of 13 days (SD 10), most commonly with ciprofloxacin (*n* = 58, 17%) and trimethoprim/sulfamethoxazole (*n* = 39, 12%).

**Fig. 1:**
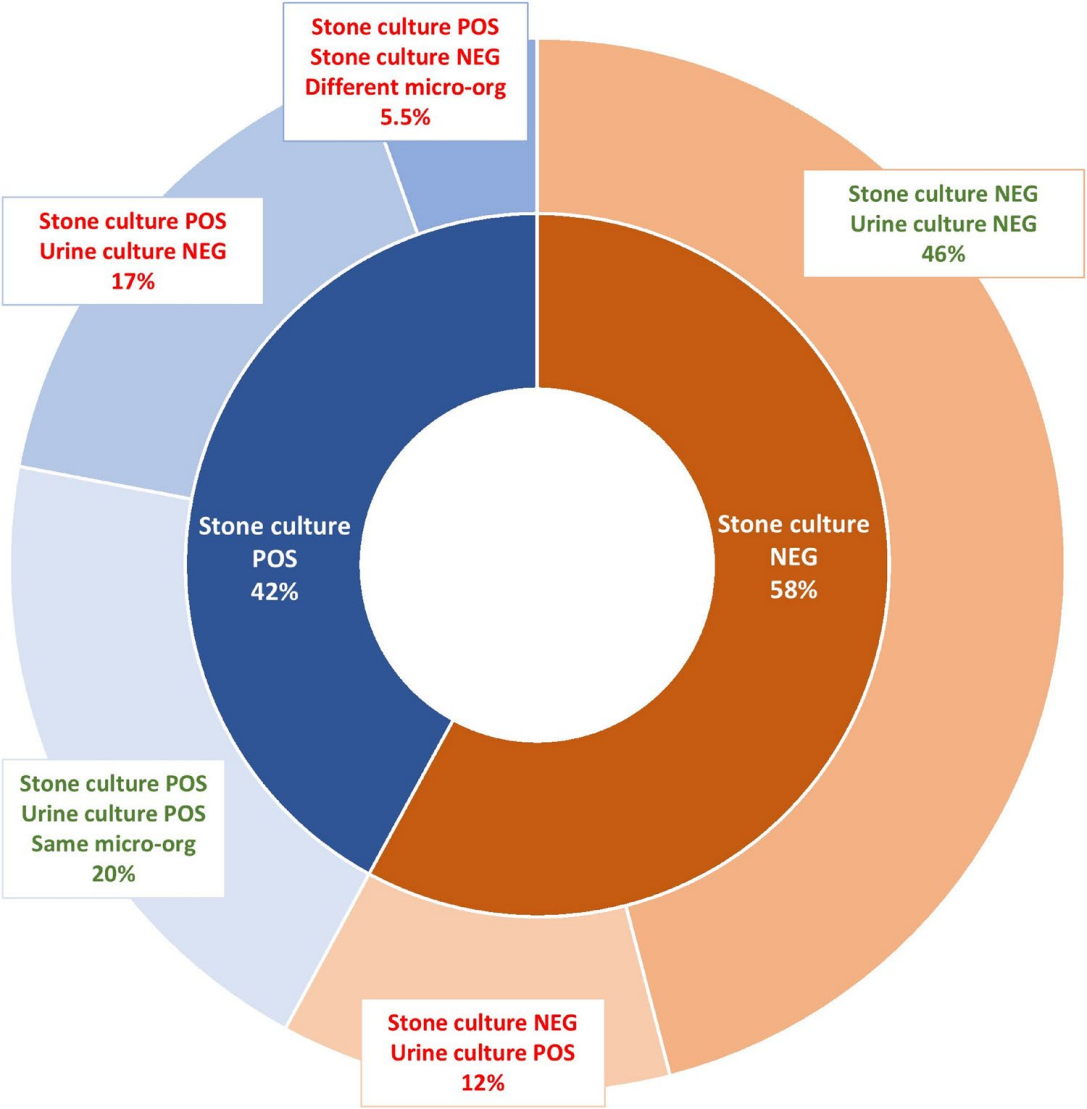
Results of perioperative stone cultures and preoperative urine tests. Green text indicates concordant results of stone cultures and urine tests. *Micro-org* microorganism

Figure 1 shows the proportion of positive and negative stone cultures and preoperative urine tests. Stone cultures were positive in 145 cases (43%) and negative in 193 (57%). Preoperative urine testing showed uropathogens in 116 cases (34%), were negative in 193 (57%) and missing in 29 (8.6%). Consequently, 309 procedures (91%) had a result for both stone cultures and preoperative urine tests.

Procedures with a positive stone culture (*n* = 145) had a negative preoperative urine test in 51 cases (35% of 145), a positive urine culture with the same microorganism in 62 cases (43% of 145), a positive urine culture with a different microorganism in 17 cases (12% of 145) and missing urine test in 15 cases (10% of 145).

Procedures with a negative stone culture (*n* = 193), had negative preoperative urine test in 142 cases(74% of 193), positive urine culture in 37 cases (19% of 193) and a missing urine test in 14 cases (7.3% of 193).

As follows, results of stone cultures and preoperative urine tests were concordant in 66% of procedures.

**Fig. 2:**
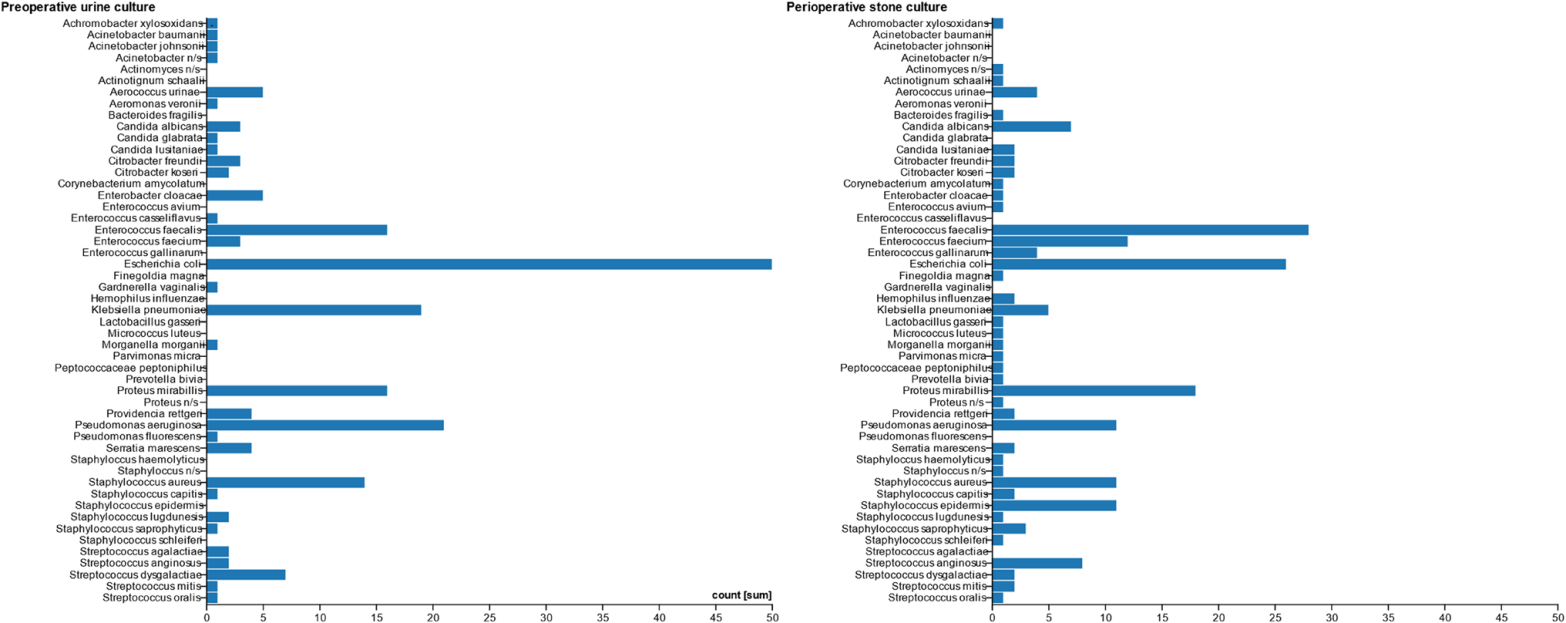
Microorganisms in preoperative urine cultures and perioperative stone cultures. *n/s* species of genus not specified

Microorganisms found in stone cultures and preoperative urine cultures are shown in Fig. 2. A wide range of 33 different microorganisms were found in stones, the most common being *Enterococcus faecalis* (28 out of 145, 18%), *Escherichia coli* (18%) and *Proteus mirabilis* (12%). This differed from the results of urine cultures, where the most prominent microorganism *Escherichia coli* was cultured in 50 out of 116 cases (43%), followed by *Pseudomonas aeruginosa* (18%) and *Klebsiella pneumoniae* (16%).

Stone composition analysis was performed in 308 procedures (91%). Table 2 presents the results of the composition analysis and the percentage of positive stone cultures per stone type. Calcium oxalate (CaOx) monohydrate (whewellite), calcium hydroxyl phosphate (carbonate apatite) and CaOx dihydrate (weddellite) were the most commonly found components of kidney stones. Percentages of positive stone cultures were high for stones containing ammonium urate (80%) and magnesium ammonium phosphate hexahydrate (struvite) (79%), but positive stone cultures were found in almost all types of kidney stones: CaOx monohydrate (whewellite) 36%, CaOx dihydrate (weddellite) 34%, basic calcium phosphate (apatite) 51%, calcium hydroxyl phosphate (carbonate apatite) 56%, calcium hydrogen phosphate dihydrate (brushite) 27%.

**Table 2:** Results of positive stone cultures per stone composition analysis result

	Component present in composition analysis *n* (% of 338 procedures)	Positive stone cultures per component *n* (%)
Calcium oxalate (CaOx)		
CaOx monohydrate (whewellite)	207 (61%)	75 (36%)
CaOx dihydrate (weddelite)	94 (28%)	32 (34%)
Calcium phosphate (CaP)		
Basic calcium phosphate (apatite)	76 (22%)	39 (51%)
Calcium hydroxyl phosphate (carbonate apatite)	130 (38%)	73 (56%)
Calcium hydrogen phosphate dihydrate (brushite)	22 (6.5%)	6 (27%)
β-Tricalcium phosphate (whitlockite)	2 (0.6%)	2 (100%)
Magnesium ammonium phosphate hexahydrate (struvite)	61 (18%)	48 (79%)
Uric acid (uricite)	39 (12%)	8 (21%)
Cystine	7 (2.1%)	1 (14%)
Ammonium urate	5 (1.5%)	4 (80%)
Calcium carbonite (aragonite)	2 (0.6%)	2 (100%)
Proteins	1 (0.3%)	0 (0%)
Xanthine	1 (0.3%)	1 (100%)
Stone analysis not performed	30 (8.9%)	12 (40%)

Table 3 shows the results of a multivariable logistic regression analysis on factors associated with a positive stone culture. A history of recurrent urinary tract infections (odds ratio [OR] 2.73, 95% CI 1.51–4.96), previous ipsilateral stone surgery (OR 1.85, 95% CI 1.05–3.26), positive preoperative urine culture (OR 3.21, 95% CI 1.80–5.74) and the presence of a preoperative nephrostomy tube (OR 2.66, 95% CI 1.24–5.67) were all statistically significant independent predictors of positive stone culture.

**Table 3:** Results of multivariable logistic regressions for associated independent variables of positive stone cultures. Bold text indicates variables with a statistically significant relationship.

Multivariable logistic regression			
*Model fit: omnibus tests of model coefficients χ*^2^ = 86.733, *p* < 0.001			
*Model calibration: Hosmer–Lemeshow goodness-of-fit test* χ^2^ = 14.030, *p* = 0.081			
Independent variable	No. procedures positive stone culture in group (%)	Exp(B) (95% confidence interval)	p value
Age			
Up to 65 years (*n* = 234)	95 (41)	REF	
65 years and older (*n* = 104)	50 (48)	1.27 (0.70–2.31)	0.4
Gender			
Female (*n* = 155)	84 (54)	REF	
Male (*n* = 183)	61 (33)	0.66 (0.37–1.16)	0.15
ASA-score			
I–II (*n* = 251)	96 (38)	REF	
III–IV (*n* = 87)	49 (56)	1.30 (0.69–2.45)	0.4
Recurrent urinary tract infections			
No (*n* = 211)	**58 (27)**	**REF**	
Yes (*n* = 127)	**89 (70)**	**2.73 (1.51–4.96)**	**< 0.001**
Previous ipsilateral stone surgery			
No (*n* = 209)	**83 (39)**	**REF**	
Yes (*n* = 129)	**62 (48)**	**1.85 (1.05–3.26)**	**0.034**
Positive preoperative urine culture			
No (*n* = 193)	**51 (26)**	**REF**	
Yes (*n* = 116)	**79 (68)**	**3.21 (1.80–5.74)**	**< 0.001**
GSS			
1–2 (*n* = 175)	73 (42)	REF	
3–4 (*n* = 163)	72 (44)	1.15 (0.67–1.99)	0.6
Preoperative nephrostomy tube			
No (*n* = 280)	**104 (37)**	**REF**	
Yes (*n* = 58)	**41 (71)**	**2.66 (1.24–5.67)**	**0.012**
Preoperative JJ-stent			
No (*n* = 298)	130 (44)	REF	
Yes (*n* = 40)	15 (38)	0.86 (0.37–1.99)	0.7

*ASA-score*  American Society of Anesthesiologists-score, *SS* Guy’s stone score

Postoperative SIRS occurred after 23 procedures (6.8%). Multivariable logistic regression found positive stone cultures were significantly associated with postoperative SIRS (OR 3.12, 95% CI 1.01–9.65) and a positive preoperative urine test was not (OR 1.60, 95% CI 0.56–4.53).

## Discussion

This study explored the practice and outcomes of kidney stone cultures obtained during percutaneous surgery, and found microorganisms in 43% of removed kidney stone fragments. Kidney stone culture results can differ significantly from preoperative urine tests and provide additional information about the presence and type of microorganisms in the urinary tract. Microorganisms were found in all types of kidney stone compositions. Finally, positive kidney stone cultures are significantly associated with postoperative SIRS.

The EAU Section of Urolithiasis (EULIS) recently conducted a systematic review and meta-analysis of 14 studies on stone cultures [15]. Among the included studies on PCNL, the reported positive stone culture rate ranged from 13 to 48%, consistent with our findings. Notably, we found concordance between stone cultures and preoperative urine testing in only 66% of cases, emphasizing the added diagnostic value of stone cultures.

Our study presents novel data on kidney stone and urine cultures in Dutch patients undergoing PCNL, identifying the spectrum of urinary tract microorganisms prevalent in the Netherlands. The most commonly isolated microorganisms from stone cultures in our cohort were *Enterococcus faecalis*, *Escherichia coli*, and *Proteus mirabilis*. In contrast, the largest study from the aforementioned systematic review, conducted in Israel, identified *Escherichia coli*, *Enterococcus spp.*, and *Pseudomonas aeruginosa* as the predominant pathogens [16]. The second largest included study was from Turkey and mostly found coagulase-negative staphylococci, *Pseudomonas aeruginosa*, and *Escherichia coli* [17]. These regional variations emphasize the importance of local microbiological data in guiding appropriate antibiotic prophylaxis and treatment for different patient populations.

A comprehensive understanding of the relationship between microorganisms and urolithiasis is essential to aid in the prevention of urolithiasis and complications during urolithiasis treatment, to which the present study contributes. The formation of ‘infection stones’ under the presence of urease-positive microorganisms (e.g., *Proteus mirabilis*, *Klebsiella* spp.) has been described extensively [6]. Bacterial urease enzymatically splits urinary urea (CO(NH_2_)_2_) into ammonia (NH_3_) and carbon dioxide (CO_2_). Subsequent hydrolysis produces two ammonium (NH_4_^+^) ions and one bicarbonate ion (HCO_3_^−^), resulting in extraordinarily high alkaline urine. The elevated urine pH level further promotes the accumulation of ammonium (NH_4_^+^), carbonate (CO_3_^2−^), phosphate (PO_4_^3−^) and magnesium (Mg^2+^), which form the typical mixture of struvite (MgNH_4_PO_4_·6H_2_O) and calcium phosphate (Ca_10_[PO_4_]_6_–CO_3_) regularly found in kidney stones in patients with urinary tract infections [1, 18, 19]. However, as the current study shows, not only magnesium ammonium phosphate hexahydrate (struvite), calcium hydroxyl phosphate (carbonate apatite) and ammonium urate stones but almost all kidney stone compositions can have positive cultures, and certainly not all microorganisms found are urease-positive. Indeed, the most cultured microorganisms from stones in this study were *Enterococcus faecalis* and *Escherichia coli*, which are both urease-negative bacteria. These remarkable findings can partly be explained by the colonization of existing kidney stones by microorganisms present in the urinary tract. However, 35% of procedures with a positive kidney stone culture had a negative urine test, and a growing body of literature describes a role for both urease-positive and urease-negative bacteria in the formation of ‘non-infection’ stones. In 1981, Cohen et al. demonstrated the presence of intracellular calcium crystals in bacteria grown in urine with strong, weak, and no urease activity [20]. Additionally, *Escherichia coli* has been shown to reduce urinary citrate levels by producing citrate lyase enzymes, which can promote CaOx crystal growth [21, 22]. Chutipongtanate et al. investigated the direct effect of *Escherichia coli*, *Klebsiella pneumoniae*, *Staphyloccocus aureus*, and *Streptococcus pneumoniae* on the growth and aggregation of CaOx crystals and found a strong promoting influence of all these bacteria [23]. Similarly, in CaP kidney stones without struvite the presence of bacterial imprints has been described, suggesting a role of microorganisms in its formation [24]. Concluding, the association of microorganisms and urolithiasis exceeds that of urease-positive bacteria and struvite stones, with open questions remaining on the additional mechanisms of microorganism-influenced urolithiasis.

The presence of microorganisms during kidney stone surgery can lead to infectious complications. The EULIS systematic review and meta-analysis compared stone cultures to preoperative bladder urine cultures for predicting postoperative sepsis and demonstrated a higher sensitivity for stone cultures (0.52 vs. 0.32), a greater positive predictive value of stone cultures (0.28 vs. 0.21), and, consistent with the present study, a higher odds ratio (5.79 (95% CI 3.58–9.38) vs. 2.30 (95% CI 1.51–3.49)) [15]. Incorporating stone cultures into local protocols can aid in tailoring antibiotic treatment for postoperative infections once results become available. Incorporating stone cultures into local protocols can aid in tailoring antibiotic treatment for postoperative infections once results become available. This is particularly relevant given the growing challenge of antimicrobial resistance in urology, for which stone cultures can play a meaningful role in antimicrobial stewardship. The observed differences between stone and urine cultures, especially in the presence of resistant organisms, underscore the importance of obtaining comprehensive microbiological data to guide targeted, narrow-spectrum antibiotic therapy. Current EAU guidelines recommend obtaining stone or renal pelvis urine cultures during PCNL whenever feasible [2]. Future research ought to explore what influence positive stone cultures should have on the use of postoperative antibiotic treatment and the management of residual stone fragments.

The cost of kidney stone cultures has not been widely reported. However, based on published estimates, the cost of a urine culture is approximately €20, while the cost of culturing prosthetic material in cases of suspected infection, which involves a comparable level of microbiological processing, is around €50 [25, 26]. These figures suggest that a stone culture represents less than 1% of the total cost of a PCNL procedure, which is estimated to range from €5,500 to €6,000 in European healthcare settings [27, 28]. 

A key strength of this study is the relatively large cohort of procedures with stone cultures and the comprehensive analysis of results by stone composition from two different centers. There were marginal differences in stone culture techniques between the two hospitals, these are unlikely to have significantly influenced the results. A key limitation of this study lies in the differing methodology used for stone cultures and urine cultures. Unlike urine cultures, which followed established thresholds for colony-forming units to define a positive results, stone cultures were interpreted based on any microbial growth, without defined cut-offs. This reflects common clinical microbiology practice, as reliable quantification is not feasible after the use of a growth medium. However, it introduces a potential bias: microorganisms isolated from stones, including low-virulence organisms or potential environmental contaminants, were considered positive. This may overestimate the clinical relevance of stone culture results. In contrast, urine cultures were interpreted using standardized quantitative criteria, and polymicrobial or non-uropathogenic growths were frequently deemed contaminated and excluded. This methodological asymmetry likely inflated the apparent discordance between urine and stone cultures and may have led to underestimation of the diagnostic value of preoperative urine cultures. These limitations should be considered when interpreting the diagnostic performance and clinical implications of stone culture findings. Further limitations of this study include its retrospective design, which led to some incomplete data. Specifically, stone culture results were available for 80% of procedures, with an additional 8.6% missing preoperative urine test results. Finally, it is important to recognize that postoperative SIRS is neither solely caused by sepsis nor definitive proof of it, as it may also arise from non-microbiological factors [5]. The relationship between stone cultures and postoperative infectious complications warrants further investigation in prospective studies incorporating systematic postoperative urine and blood cultures. The absence of such standardized follow-up in the present study limits the ability to assess the true predictive value of stone cultures for infection and their impact on antibiotic management.

## Conclusions

43% of kidney stones removed via PCNL had microbiological growth. In 47% of these procedures, preoperative urine testing had been negative or positive with a different microorganism. A wide range of microorganisms was found in kidney stone cultures, not limited to urease-positive bacteria. Positive stone culture results are not limited to so-called infection stones (e.g., struvite and carbonate apatite) but are regularly found across all types of stone composition.

However, the clinical significance of stone cultures, particularly in terms of guiding antibiotic therapy or predicting infectious outcomes, remains to be established. Future studies should incorporate standardized postoperative urine and blood cultures, predefined antibiotic strategies, and clinical follow-up protocols to better define the role of stone cultures in patient management. As an observational and hypothesis-generating study, this work lays the groundwork for further research into how intraoperative stone cultures could contribute to tailored perioperative (antibiotic) care.

## Data Availability

Data has not been published but it available on request.

## Abbreviations

CaOx: Calcium oxalate
CaP: Calcium phosphate
CT: Computed tomography
ECIRS: Endoscopic combined intrarenal surgery
EAU: European Association of Urology
GSS: Guy’s Stone Score
JJ: Double-J ureteral stent
NT: Nephrostomy tube
PCNL: Percutaneous nephrolithotomy
SIRS: Systemic inflammatory response syndrome
URS: Ureterorenoscopy
